# A Dynamic Analysis of the Demand for Health Care in Post-Apartheid South Africa

**DOI:** 10.3390/nursrep11020045

**Published:** 2021-06-17

**Authors:** David Mhlanga

**Affiliations:** Department of Accountancy, The University of Johannesburg, Auckland Park, P.O. Box 524, Johannesburg 2006, South Africa; dmhlanga67@gmail.com

**Keywords:** demand for health, nursing, post-apartheid, South Africa

## Abstract

The study aimed to investigate the drivers of demand for healthcare in South Africa 26 years after democracy. The pattern healthcare demand by households in South Africa is that most households use public healthcare services particularly public clinics compared to private and traditional healthcare facilities. Using conditional probability models, the logit model to be more specific, the results revealed that households head who is unemployed, households who do not have a business, households who were not receiving pension money, had a greater probability of demand for public healthcare institutions. On the other hand, being male, being White, Indian and Coloured, being a property owner and being not a grant beneficiary, reduces the probability of demand for public healthcare facilities in South Africa. As a result, the study recommends more investment in public healthcare but more in public clinics in South Africa due to the high percentage of households using these services. Also, the government must consider investing more in the maintenance and improvement of the welfare of nurses in the country considering the huge role they play in the delivery of healthcare to the citizens.

## 1. Introduction and Background

As a nation becomes wealthy, households and individuals will start to take life more seriously which will lead them to demand more healthcare services. The rise in demand for healthcare will lead to the escalation of healthcare spending [1,2]. The rise in the number of elderly people in a nation also results in the rise in demand for healthcare services as these people take more consistency of their health. It is estimated that spending for global health can rise from US 8 trillion in 2018 to US$ 18 trillion in 2040 [2]. It is also projected that approximately 9% of GDP globally will be allocated to health in 2040 as propounded by the Institute for Health Metrics and Evaluation (IHME) [2]. The major feature of demand for healthcare is the actual consumption of the products and services by an individual who is facing illness or injury [3]. The factors such as the level of income, the cost of care, education levels of the households, the societal norms, values, and traditions as well as the quality of the services provided influences consumption levels of these services and products [4].

Many countries especially developing nations are putting more effort to try and promote healthcare utilization as one of the important policies to improve access to healthcare and to meet goal three of the sustainable development goals [3]. Goal three of the sustainable development goals outlines that countries should ensure healthy lives and promote wellbeing for all ages.

Countries are pursuing to achieve this goal among other targets such as ensuring universal access to sexual and reproductive healthcare services, which include family planning, information, and education, as well as the integration of reproductive health into national strategies and programs. Also, other targets involve issues to do with universal health coverage, including financial risk protection, access to quality essential healthcare services and access to safe, effective, quality, and affordable essential medicines and vaccines for all by 2030 [5]. Developing countries should not focus on initiatives that improve physical access only without giving due diligence to the pattern of healthcare utilization associated with the demand side [5]. Researchers are arguing that in emerging economies citizens continue to suffer from poor healthcare services due to insufficient healthcare funding and the general under-utilization of healthcare services.

It is believed that in n developing countries, the under-utilization of healthcare services is a cause for concern caused by a plethora of factors such as lack of adequate supply of healthcare services [3,6]. However, in situations where these services are present, utilization of healthcare services has been low due to several barriers from the demand side associated with travelling cost, quality, and the cost of treatment [7,8]. The World Bank Group (WBG) believe that access to health is a vital component in fighting global challenges such as poverty [9]. Also, the WBG [9] believes that the health and poverty relationship is bidirectional and financial. It is generally believed that poverty is a major cause of ill health and a barrier to accessing health care when individuals need it [9,10]. The financial part of the relationship comes in because the poor cannot afford to purchase those things that are needed for good health, including enough quality food and health care.

It was also outlined that the relationship of poverty, health and health access is also related to other factors associated with poverty, like lack of information on appropriate health-promoting practices or lack of voice needed to make social services work for them [11,12]. In South Africa, health care reform has been the heart of the country’s development agenda, the government of South Africa is striving to improve access to health care for the poor and marginalized through expanding the healthcare facility network and abolishing user fees for primary healthcare [13]. However, despite these efforts’ healthcare access remains polarized, unequal and to some extend unfair [13].

In 2018, StatsSA general household survey (GHS) indicated that nationally, 71.5 per cent of households prefer public clinics, hospitals, or other public institutions when they are ill or involved in an accident, while 27.1 per cent of households consult a private doctor, private clinic, or hospital first whenever they are ill. Only 0.7 per cent of households consult a traditional healer first whenever they are ill [14]. These statistics show that more households in South Africa use public healthcare institutions compared to private healthcare centres. However, the department of health in 2017 highlighted that the private sector spent 4.4 per cent of GDP on health but only provides care to 16 per cent of the population, while the public sector spent 4.1 per cent of GDP on health but had to provide care to 84 per cent of the population [15,16].

With this information and the fact that one of the policy priorities of nations globally is to improve the health status of the population, there should be an investigation of the factors that affect the demand for health care services and products directly or indirectly in South Africa. There are various studies conducted that tries to investigate the demand for health for instance a study conducted in Ethiopia by Wellay et al. [3] indicated that being educated, cost of health care and distance to the health facility increases the probability of an individual demanding healthcare services. In another study conducted in Kenya, Nyambura [17] discovered that the age of the household and the quality of care was important in influencing demand for a healthcare facility. Another study by Zhou et al. [2] that examined the determinants of healthcare spending among the emerging economies found out that the growth of the economy and the number of ageing populations can induce healthcare costs in emerging nations. The study also discovered that factors like technological advancement, agricultural activities, and industrialization can also influence healthcare spending. Another important factor that Zhou et al. [2] discovered was that there should be an effort on financial stability to ensure that there is universal access to healthcare at the same time activities as broadening of the revenue base through various means such as agriculture, industrialization is intensified. Ali & Sayed, [18] also investigated the factors that affect government healthcare spending in Gulf Council (GCC) nations. The study discovered that average healthcare spending per capita in GCC nations have a significant relationship with the revenue of the government, the population size, and the public debt of the nation. Motivated by these findings the current study intends to use the 2018 general household survey data in South Africa to investigate the factors that influence households to demand public healthcare. This can help to find appropriate measures to address the imbalances associated with access to healthcare services and to reveal some of the specific barriers in the process.

### 1.1. The Health Care Sector in South Africa

In South Africa, private and public health systems exist in parallel. The public system serves most of the population even though there are reports that the public health sector is sometimes underfunded and understaffed [19]. The first hospital in South Africa was started at Cape of Good Hope in 1652 as a temporary tent meant to provide care for sick sailors of the Dutch East India Company affected by typhoid and scurvy [20]. In the year 1656, a permanent hospital was completed in South Africa and convalescent soldiers provided care up to 1700 when the first matron and male nurse were appointed to ensure cleanliness in the hospital and to supervise bedside attendants [20,21]. Many hospitals were built in South Africa in 1807 to meet the increase in demand for healthcare. These hospitals were founded in Port Elizabeth, King Williamstown, Graham’s town, and Queenstown in the Eastern Cape [19,21].

In the period which stretches from 1912 to 1944, there was an improvement and introduction of several activities in the health department in the country. For instance, the South African military recognized the importance of military nursing in the Defense Act in 1912 and the first nursing journal was introduced in 1913 [19] (Coovadia et al., 2009). Also, 1935, marked the introduction of the first diploma courses which enabled nurses to train as tutors at the University of Witwatersrand and the University of Cape Town [15,21]. Independent nursing councils and Nursing Associations were later created as a response to the establishment of independent states and homelands in South Africa. These include Transkei, Bophuthatswana, Venda, and Ciskei.

However, under the post-Apartheid dispensation, these independent nursing councils and associations were all merged to form one organization, the Democratic Nursing Organization of South Africa (DENOSA) [19,22]. Currently, in South Africa, there are more than 400 public hospitals and more than 200 private hospitals [15,19]. The provincial health departments manage the larger regional hospitals directly while smaller hospitals and primary care clinics are managed at the district level. Moreover, the national department of health manages 10 major teaching hospitals directly. Likewise, in South Africa, the Chris Hani Baragwanath Hospital is the third-largest hospital in the world, and it is in Johannesburg [15].

### 1.2. The Use of Healthcare Facilities in South Africa

**Figure 1:**
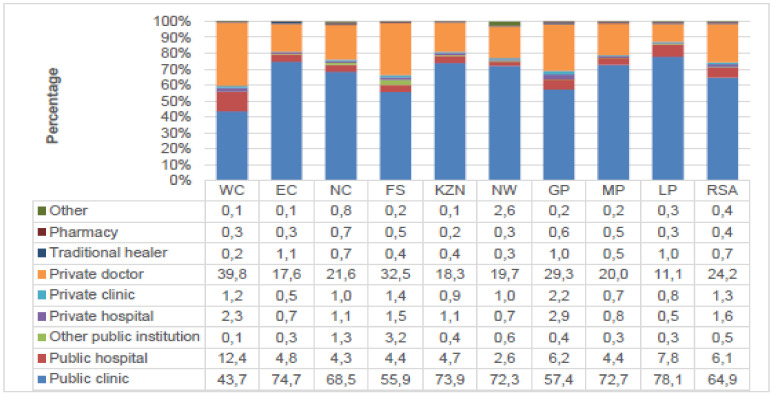
Percentage distribution of the type of healthcare facility consulted first by the households when members fall ill or get injured by province. Source: StatsSA [14].

In South Africa, households have different tastes when it comes to the choice of a healthcare providing facility. Figure 1 below gives a percentage distribution of the type of health-care facility consulted first by the households when members fall ill or get injured by province.

Figure 1 is showing the percentage distribution of the health care facility consulted first by the household when they need help. The information from Figure 1 indicated that nationally 71.5 per cent of households prefer public clinics, hospitals, and other public institutions when they require help. About 27.1 per cent of households go to private healthcare facilities like private doctor, private clinic, or hospital when they require any healthcare-related service. Those who responded that they would go to a traditional healer were 0.7 per cent. From all the healthcare facilities, public clinics were the facilities mostly used by households in South Africa in all the provinces. For instance, 78.1 per cent of the households in Limpopo, 74.7 per cent in the Eastern Cape indicated that they consult public clinics first when they are ill or get injured. This shows that In South Africa nurses in public facilities are doing a lot in the provision of healthcare to the citizens more than any other workers in the sector.

## 2. The Theoretical Framework

According to Grossman [7] health is regarded as a durable capital good which is inherited and depreciates over time. The demand for healthcare is regarded as a derived demand for health. Healthcare is demanded as a way for consumers to attain a larger stock of health capital [23]. The demand for health is different from other products because with health individuals allocate resources in a way to both produce and consume health [24,25,26,27]. The Michael Grossman demand for health model explain the demand for health.

### Michael Grossman Model

The Grossman model is a model propounded by Michael Grossman in 1972 which explains the demand for health and medical care. This model was first published in Michael Grossman monograph of 1972 “titled a theoretical and empirical investigation”. Grossman’s model is premised on the interaction of the demand function for health and the production function for health. Also, Michael Grossman views health as a capital durable good, inherited by an individual but depreciates for a while [7,23]. On the other hand, investment in health is in the form of medical care purchases among other inputs, while depreciation is viewed as a natural deterioration of health over time [7]. In the model, health will enter the utility function directly as a good which people drive pleasure from and indirectly as an investment which allows more health time to be available for market and non-market activities [7]. As a result, this study is investigating the demand for healthcare where people are taken as consumers.

## 3. Empirical Literature Review

There are quite many studies done to try and understand the demand for health, Kirdruang [28] investigated the determinants of use of public hospitals utilization in Thailand. Using the multinomial logistic regression, the study discovered that income, amount of co-payment, family size and age of the household head were the factors significantly influencing the demand for public healthcare centres. In another study, 30. Kansiime et al. [29] assessed the factors influencing the patient to choose a healthcare Mburo National Park one of the districts of Uganda. Using the multinomial logit model, the study discovered that religion, education level and availability of drugs and hospital equipment were the significant factors in influencing the demand for healthcare facilities. Also, Kukla et al. [30] examined the influence of transport cost on household to choose a healthcare provider from selected African countries and South Asian countries which include Gambia, Kenya, Pakistan, and India for malaria patients. The medical healthcare facilities were grouped into three groups, self-treatment, private healthcare, and public health care facilities. Using the multinomial logit model, the study concluded that severity of illness, direct cost incurred such as consultation fees, costs of medication were the significant factors influencing the choice of the healthcare provider in both Asian and African countries.

Moreover, Galal & Al-Gamal, [31] carried out an analysis to investigate the factors influencing households to choose healthcare provider in Egypt. The study used logistic regression to critically assess the factors influencing the probability of a household choosing a healthcare provider. The study concluded that the factors influencing the choice of a healthcare provider by the households include distance to the nearest health care, reputation of the healthcare facility and quality of service offered. These factors influence the probability of a household choosing one healthcare provider from the other Huang & Gan, [1] also assessed the factors that affect the demand for healthcare services in China using the China Health and Nutrition Survey. The study found out that increased cost of sharing is one of the factors that decrease the outpatient medical care utilization and expenditures without a decrease in inpatient utilization and expenditures. The study by Huang & Gan, [1] also discovered that low-income and middle-income households and households with limited severe medical conditions are more sensitive to prices. Osakede, [32] also investigated the facility determinants of healthcare demand in Nigeria. The study found that the presence of nurses in various facilities can help to increase the number of visits to healthcare facilities.

Fe et al. [33] investigated the agency problem between patients and doctors in rural China. The study found out that there was no correlation between doctor competency and patient’s health care utilization. The study discovered that household perceptions of quality are a critical determinant of care-seeking behaviour. However, the major problem is that many patients cannot recognize the competency of doctors. As a result, the study also found out that there is no relationship between doctor competency and perception of quality. Harvey et al. [34] also evaluated whether antenatal supply-side and demand-side interventions can increase the number of women who had four or more antennal care visits and health care deliveries in Kenya. The results showed that combined antenatal and insurance interventions can motivate an increase in antenatal care visits and healthcare deliveries. The study also discovered that women prefer private health care facilities for delivery compared to public health care facilities. The next section will show the methods and data used in this study.

## 4. Methods and Data

The study used data from the 2018 general household data of South Africa. From this data, relevant data were extracted from the data set to fulfil the objective of the study.

### 4.1. Dependent Variable

The dependent variable is dichotomous, that is either the household chooses public healthcare or not represented by a 0 or 1, respectively. The dependent variable is generated from the question which was asking participants on which medical healthcare institution they will go to first when they are ill or when they are involved in an accident (public, private, traditional). The data was recorded to appear in a binary form. The situations where the household chooses public healthcare institution for help was named 1 while in circumstances where the household chooses other healthcare institutions will take 0.

### 4.2. Independent Variables

Guided by the works of Andersen’s Behavioral Model [35], Grossman’s Model of Demand for Health [7], and the literature on the determinants of demand for healthcare facility the following independent variables were used in the study. Please see Table 1.

The table above is showing all the independent variables used in the study. The model tested for multicollinearity and the results indicated that the model is free from multicollinearity since all the independent variables had a Pearson product-moment correlation coefficient, with a value of less than 0.8 in absolute terms. Kennedy [36] outline that, for non-continuous variables, a value of 0.8 and above in absolute terms in one of the correlation coefficients indicates a high correlation between two independent variables. The section below is giving us the empirical model used in this study.

### 4.3. Empirical Model

Maximum Likelihood Estimation (MLE) as outlined by Green and Silverman, [37], Maddala et al. [38]. The MLE results to model with a variable shown in equation 1 below:

(1) $$Y_{i}=\left\{\begin{array}{c} 1\;if\;Y_{i}>0\\ 0\;otherwise \end{array}\right.$$

In Equation (1) above shows the unobserved variable of $Y_{i}$ which becomes a latent variable given as $Y_{i}^{*}$ expressed in Equation (2) below:

(2) $$Y_{i}^{*}=\beta_{0}+\overset{k}{\underset{j=1}{\sum }}\beta_{i}X_{ij}+\mu_{i}$$

Equation (2) above can be expressed as a logit or probit model depending on the distribution of the error term $\mu_{i}$ [39]. In this paper, the logit model was used which is an extension of the probit model [39].

The logistic probability function is specified clearly in Equation (3) below:

(3) $$P_{i}=F\left(Z_{i}\right)=F\left(\alpha +\overset{n}{\underset{i=1}{\sum }}\beta_{i}X_{i}\right)=\frac{1}{1+e^{-Zi}}$$

From Equation (3), $P_{i}$ is the probability that an individual will choose public healthcare or not given $X_{i}$. Also, $X_{i}$ represents the $i^{th}$ explanatory variables and n is the total number of explanatory variables and e denotes the base of natural logarithms which is approximately equal to 2.718. $\beta_{i}$ and α, are parameters to be estimated.

The study used the logit model because of the following advantages. The probit model has a restrictive assumption that the error term should be normally distributed [40]. As a result, the logit model is generally flexible and it is easy to use from the mathematical point of view [39]. Also, the results generated from the logit model are more meaningful in interpretation compared to other models [40]. 

The results from the estimation of the drivers of demand for public health care in South Africa from the 2018 GHS data using the logit model are presented in Table 2 below. 

The results showed that sex of household head, the race of the household head, age of household head, property ownership, being a grant beneficiary, being a pension receiver, being an employee receiving salaries/wages, business ownership that is receiving income from the business were the significant factors influencing household to use public health facilities. However, from these factors, some influenced the households to use more of the public health facilities while others reduced the use of public health facilities. Starting with the gender of the household head, the variable was significant at a 1 per cent level of significance with a *p*-value of 0.004. The results from the logistic regression further showed that there was a positive relationship between gender and the use of public health facilities. The meaning of the result was that being female increases the probability of use of public health facilities compared to males. The odds of use of public health facilities are 1.131 higher for females compared to males. This indicates that women in South Africa use more public health facilities like public clinics and public hospitals compared to males.

Also, the variable race/population group was significant at a 1 per cent level of significance (*p* < value, 0.000). Taking the black population group as a reference category the results revealed that, being Coloured, Indian and White reduces the probability of a household using public health facility. The odds of using public health facilities were lower for Whites which was 0.056 compared to Indians with 0.128, Coloureds 0.447 and Blacks with 4.390. The results further indicate that being black in South Africa increases the probability of the household using public health facilities compared to other population groups. Furthermore, the results revealed that the age of the household head was a significant variable (*p* < value, 0.000) influencing the demand for the use of public health facilities in South Africa. The variable influences the probability of demand for the use of public health facilities negatively. The odds of use of public health facilities were 0.987. The meaning of the negative sign was that with a unit increase in the age of a household head, the demand for services provided by public health facilities declines greatly. This may be because as individuals grow, the income also tends to rise with an increase with opportunities and experience brought up by age. The rise in income will motivate these households to demand more private health facilities compared to public healthcare facilities.

Moreover, the variable house ownership was significant in influencing the demand for use of public healthcare facilities. The results indicated that owning a house by the households in South Africa reduces the probability of demand for use of public health facilities. This means that households who are owners of properties demand fewer services offered by public health facilities compared to services offered by private health facilities. The variable was significant at the 10 per cent level of significance with a *p*-value of 0.083, while the odds ratio was 0.928. The variable grant was significant at a 1 per cent level of significance with a *p*-value of 0.000. The variable had a negative relationship with the demand for the use of public health facilities. Non- grant recipients had less probability of using public health facilities compared to grant receivers. Non-grant recipients had less probability of using public health facilities compared to grant receivers. The meaning of the negative sign was that non-grant recipient households had less probability of using public health facilities compared to households who were grant recipients. The odds ratio for none -grant recipients was 0.189 less compared to grant recipient households. Receiving grants increases the probability of using public health facilities compared to none- grant recipients.

Also, the variable pension was significant and positive in influencing the probability of using public health facilities. The variable was significant at a 1 per cent level of significance with a *p*-value of 0.000 and an odds ratio of 3.272. The meaning of the results was that households who are not recipients of pensions had a higher probability of using public health facilities compared to households who were recipients of pensions. Non-pension receivers had a 3.272 higher probability of using public health facilities compared to pension receivers. The variable salaries/wages/commission was a significant variable in influencing the probability of using public health facilities. The variable was significant at a 1 per cent level of significance with a *p*-value of 0.000. The results indicated that households who do not have income from salaries or wages had a higher probability of using public health facilities compared to households with income from wages or salaries. Households without income from wages/salaries had had a higher probability by 2.633 compared to a household with income from wages/salaries.

The other significant variable was income from the business, the variable was significant at a 1 per cent level of significance (*p* < value, 0.000) with a positive influence on the probability of using public health institutions. The variable showed that households without income from business had a higher probability of using public healthcare institutions compared to households with income from businesses. The odds ratio for households without income from the business was 1.401 higher compared to households with income from a business.

## 5. Conclusions and Policy Recommendations

The purpose of the study was to assess the factors that influences households in South Africa to use more public healthcare like public clinics compared to private and traditional health centres. The pattern of choice of healthcare by households in South Africa showed that most households prefer public healthcare centres like public clinics compared to other healthcare facilities. To critically assess the factors that influences household to demand public health facilities the study used the logit model. The logit model was used to understand the factors that influence the probability of a household to demand public healthcare centres more compared to others. The results showed that sex of household head, the race of the household head, age of household head, property ownership, being a grant beneficiary, being a pension receiver, being an employee receiving salaries/wages, business ownership that is receiving income from the business were the significant factors influencing household to use public health facilities. However, from these factors, some influenced the households to use more of the public health facilities while others reduced the use of public health facilities. For instance, being a grant beneficiary increases the probability of a household using public healthcare facilities compared to households who were not receiving grants. Therefore, the study recommends more investment in public healthcare centres but more in public clinics in South Africa. This is because more households prefer public clinics compared to other centres for health-related services. The government of South Africa needs to invest more in the welfare of nurses who work in public health clinics across the country as they are the people who put more effort into availing healthcare services to the people in the country.

## Figures and Tables

**Table 1:** Description of independent variables used in the study.

	Variable	Description
X1	Gender	This variable is a dummy variable where 1 = male and 0 otherwise. The variable is expected to be + influence on the choice of public health care centres for women and negative for private health care institutions.
X2	Age	This variable is a continuous variable that explains the number of years of the individual. The variable is expected to be a positive influence on access to public and private health facilities Age (years).
X3	Household size	Explains the number of people in the household, the variable is expected to have a positive influence on the choice for public institutions and traditional institutions while negative on the private institution.
X4	Net household income per month in Rand	This variable is described as the amount of money income earned by the household per month. The variable expected to have a positive or negative.
X5	Own house	The variable is a dummy variable where 1 is described as owning a house while 0 otherwise. The variable can have a negative or positive influence on the choice of a healthcare provider
X6	Grants	Grants is a dummy variable that takes the value of 1 when a household receives grants and 0 otherwise.
X7	Salaries/wages/commission	Salaries/wages/commission is a dummy variable where the variable takes the value of 1 if the household receives salaries/wages while 0 otherwise. The variable is expected to have a positive or negative influence.
X8	Remittances	Remittances is a dummy variable that takes the value of 1 if the household receives. The variable is expected to have a positive or negative influence
X9	Pensions	Pensions is a dummy variable that assumes the value of 1 if the household receives pension and 0 otherwise. The variable is expected to have a positive or negative influence.
X10	Income from a business	Income from a business is the total income a household receives from a business

Source: Author’s Analysis.

**Table 2:** Logit results, use of public health facilities.

Variables in the Equation							
		B	S.E.	Wald	df	Sig.	Exp(B)
Step 1a	Sex of household head (1)	0.123	0.043	8.339	1	0.004	1.131
	Population group of the household head			1400.425	3	0.000 ***	
	Population group of household head (1)	−0.804	0.064	159.474	1	0.000 ***	0.447
	Population group of household head (2)	−2.060	0.121	288.422	1	0.000 ***	0.128
	Population group of household head (3)	−2.883	0.087	1102.499	1	0.000 ***	0.056
	Age of household head	−0.013	0.002	63.049	1	0.000 ***	0.987
	Household size	−0.009	0.011	0.703	1	0.402	0.991
	House ownership (1)	−0.075	0.043	3.003	1	0.083 *	0.928
	Grant (1)	−1.665	0.054	940.234	1	0.000 ***	0.189
	Pensions (1)	1.185	0.093	161.629	1	0.000 ***	3.272
	Salaries/wages/commission (1)	0.968	0.055	315.233	1	0.000 ***	2.633
	Income from a business (1)	0.337	0.060	31.804	1	0.000 ***	1.401
	Remittances (1)	−0.091	0.066	1.915	1	0.166	0.913
	Constant	1.479	0.177	69.604	1	0.000	4.390

a Variable(s) entered on step 1: Sex of household head, Population group of household head, age of household head, household size, House ownership, Grants, Pensions, Salaries/wages/commission, Income from a business, Remittances. Model Summary Step, −2 Log likelihood17001.588a, Cox & Snell R Square 0.253, Nagelkerke R Square 0.376. Omnibus Tests of Model Coefficients, Chi-square Step, 6016.607, Block, 6016.607, Model, 6016.607, df, 12, Sig, 0.000. (Significant, 1 percent ***, 5 percent **, 10 percent *). Source: Author’s Manipulation.

## Data Availability

Data available in a publicly accessible repository that does not issue DOIs. Publicly available datasets were analyzed in this study. This data can be found here http://www.statssa.gov.za/, accessed on 4 March 2021.
